# Serum NT-proBNP Levels Are Not Related to Vitamin D Status in Young Patients with Congenital Heart Defects

**DOI:** 10.1155/2016/3970284

**Published:** 2016-02-03

**Authors:** E. Passeri, R. Rigolini, E. Costa, C. Verdelli, C. Arcidiacono, M. Carminati, S. Corbetta

**Affiliations:** ^1^Endocrinology Unit, IRCCS Policlinico San Donato, 20097 San Donato Milanese, Italy; ^2^Laboratorio di Chimica Clinica, IRCCS Policlinico San Donato, 20097 San Donato Milanese, Italy; ^3^Laboratory of Molecular Biology, IRCCS Policlinico San Donato, 20097 San Donato Milanese, Italy; ^4^Pediatric Cardiosurgery, IRCCS Policlinico San Donato, 20097 San Donato Milanese, Italy; ^5^Endocrinology Unit, Department of Biomedical Sciences for Health, University of Milan, IRCCS Policlinico San Donato, 20097 San Donato Milanese, Italy

## Abstract

*Context*. Hypovitaminosis D frequently occurs in early life and increases with age. Vitamin D has been suggested to influence cardiac performance and N-terminal-pro-type B natriuretic peptide (NT-proBNP) release in adults with heart failure.* Objectives*. To assess the vitamin D status and the impact of hypovitaminosis D on circulating NT-proBNP levels in young patients with congenital heart defects (CHD).* Design and Patients*. This cross-sectional study included the assessment of serum 25-hydroxyvitamin D (25OHD), parathyroid function markers, and NT-proBNP levels in a series of 230 young in-patients (117 females, 113 males; 6.4 (4.0–9.1) years (median, interquartile range)) with CHD.* Results*. Serum 25OHD levels <20 ng/mL were detected in 55.3% of patients. Optimal 25OHD levels (>30 ng/mL) occurred in 25% of patients. Serum 25OHD levels inversely correlated with age (*r* = −0.169, *P* = 0.013) and height standard deviation score (*r* = −0.269, *P* = 0.001). After correction for age, 25OHD negatively correlated with serum PTH levels (*β* = −0.200, *P* = 0.002). PTH levels above the upper quartile (44 pg/mL) occurred in 32% of hypovitaminosis D patients. Serum NT-proBNP levels were not correlated with 25OHD and PTH levels.* Conclusions*. Half of the young CHD patients were diagnosed with 25OHD deficiency and a third of hypovitaminosis D patients experienced hyperparathyroidism. Nonetheless, serum NT-proBNP levels were not associated with hypovitaminosis D as well as hyperparathyroidism.

## 1. Introduction

Congenital heart defects (CHD) occur in 0.5–0.9% of newborns and survival is substantially improved in the last decades [1–5]. From a pathophysiologic standpoint, CHD may be characterized by (1) increased volume overload (i.e., defects characterized by left-to-right shunt, such as ventricular septal defect, patent ductus arteriosus, truncus arteriosus, atrial septal defect, and atrioventricular septal defects); (2) pressure overload involving the left ventricle (i.e., aortic stenosis and aortic coarctation) or the right ventricle (i.e., tetralogy of Fallot and pulmonary stenosis); (3) complex cyanotic CHD (i.e., univentricular heart and transposition of the great arteries).

The N-terminal-pro-type B natriuretic peptide (NT-proBNP) is 76 amino acids derived from cleavage of a prohormone of 108 amino acids synthesized and released by cardiomyocytes. NT-proBNP can be used as an adjunctive marker in the integrated screening, diagnosis, management, and follow-up of children with heart failure caused by various acquired and congenital heart diseases [6]. The natriuretic peptides, NT-proBNP and BNP, correlate with various indexes of disease severity in children with congenital heart defects. The measurement of the circulating cardiac biomarkers BNP and NT-proBNP is now recommended by international guidelines [7, 8].

Hypovitaminosis D is prevalent worldwide: around 37% of studies evaluating the vitamin D status in various populations reported mean values below 20 ng/mL [9]. Pediatric patients with congenital heart defects might be a population at high risk of hypovitaminosis D due to their chronic disease and to the reduced exposure to sunlight. Vitamin D is key component of the calcium metabolism. Calcium and the calciotropic hormones, vitamin D and parathormone (PTH), exert direct and indirect effects on cardiomyocytes. Calcium dyshomeostasis associated with hypocalcemia, hypovitaminosis D, and secondary hyperparathyroidism can lead to the development of dilated cardiomyopathy with life-threatening consequences in both infants [10, 11] and adults [12]. Moreover, cardiomyocytes express both the vitamin D receptors and the PTH receptors (PTHR1), and studies in rodents have shown that vitamin D protects against cardiac hypertrophy and myocardial dysfunction [13–16]. In line with experimental data, vitamin D deficiency has been associated with a poor prognosis in adult patients with heart failure [17], and circulating PTH levels are related with an increased risk of cardiovascular events and mortality [18–20].

In the present study, we tested the hypothesis that vitamin D status might influence cardiac performance and therefore the circulating NT-proBNP levels in young patients with congenital heart defects. We investigated (1) the vitamin D status and (2) the correlations of calcium metabolism markers with serum NT-proBNP levels in a consistent series of young patients with various congenital heart defects.

## 2. Patients and Methods

### 2.1. Study Population

Two hundred thirty young patients (117 females, 113 males; 6.4 (4.0–9.1) years (median age, interquartile range)) with CHD, referred to the Policlinico San Donato Pediatric Cardiosurgery between January 2007 and January 2009, were consecutively enrolled. All patients were studied at the admission to be evaluated for congenital heart defects and to undergo open or transcatheter surgical correction when haemodynamically significant CHD was diagnosed. In particular, 153 patients harboured defects determining increased volume overload (i.e., defects characterized by left-to-right shunt, such as ventricular septal defect, patent ductus arteriosus, truncus arteriosus, atrial septal defect, and atrioventricular septal defects); 62 patients had heart defects with pressure overload involving the left ventricle (i.e., aortic stenosis and aortic coarctation) or the right ventricle (i.e., tetralogy of Fallot and pulmonary stenosis); and 15 patients were affected with complex cyanotic CHD (i.e., univentricular heart and transposition of the great arteries).

Anamnestic and anthropometric parameters were recorded. Height was evaluated by height standard deviation score (HSDS) related to Italian growth curves for children aged 2–20 years [21]. HSDS for infants aged 0–2 years was calculated by using the Kabi-Pharmacia Growth Calculator, based on the British standards. Weight was evaluated by Weight-to-Height Index (WHP) expressed as the percentage of the median of the British standards using the Kabi-Pharmacia Growth Calculator. Clinical data are shown in Table 1. Exclusion criteria were considered overt cyanotic status, acute or chronic kidney failure, liver failure, infective and inflammatory diseases, acquired or metabolic cardiomyopathy, concurrent treatment with diuretics, amiodarone and steroids, ongoing calcium and/or vitamin D supplementation in the last 6 months, overt hypoparathyroidism, and overt or mild hypothyroidism.

Informed consent for personal data and blood collection was obtained by parents of all patients. The study was approved by the local ethical committee.

### 2.2. Measurements

Venous blood sampling was collected the day before the diagnostic procedure after an overnight fasting, and biochemical and hormonal markers were measured in all patients. Serum calcium, phosphate, magnesium, and creatinine were measured by routine assays. Serum calcium levels were corrected for the serum albumin concentrations according to the formula: serum total calcium (mg/dL) − 0.8 × [serum albumin (g/dL) − 4.0]. Serum albumin concentrations were within the normal range in all patients indicating the absence of malnutrition. Ionized calcium concentrations were assayed by Liquichek Blood Gas (IL Synthesis Series, Bio-Rad Laboratories, Segrate, MI, Italy). Serum 25OHD concentrations were measured by a chemiluminescent assay (LIAISON test, DiaSorin Inc., Stillwater, MN, USA) with mean intra- and interassay coefficients of variation (CV) of 4.5% and 7.5%, respectively. Serum NT-proBNP and PTH were measured by Electrochemiluminescence on an Elecsys 2010 (Roche Diagnostics, Mannheim, Germany).

### 2.3. Statistical Analysis

Continuous parameters following a nonnormal distribution were presented as median and interquartile range (IQ). Normally distributed parameters were presented as mean ± standard deviation; categorical data were given as percentages. Based on serum 25OHD concentrations, we formed four categories according to widely used cut-off values [22–24]: severe deficiency, less than 10.0 ng/mL; moderate deficiency, 10.0–19.9 ng/mL; insufficiency, 20.0–29.9 ng/mL; and 25OHD optimal range, 30.0–100.0 ng/mL. For nanomoles per liter, multiply by 2.496. Serum 25OHD, PTH, and NT-pro-BNP concentrations were logarithmically transformed before being used in parametric procedures. Comparisons among CHD groups were performed by analysis of variance (ANOVA) with *P* for linear trend for continuous parameters or ANOVA on ranks for nonparametric data. A *χ* ^2^ test was performed for categorical variables. Simple correlation analyses (Pearson or Spearman correlations where appropriated) and multiple linear regression analyses including several independent variables were performed to examine whether 25OHD levels were associated with NT-pro-BNP levels. A *P* value <0.05 was considered statistically significant. Data were analysed using SPSS 22.0 statistical package (SPSS 22.0 Inc., Chicago, IL).

## 3. Results

### 3.1. Vitamin D Status in Young CHD Patients

Assessment of serum 25OHD levels in all CHD patients showed a high prevalence of vitamin D deficiency: serum 25OHD levels < 20 ng/mL were detected in 55.3% of young CHD patients and severe vitamin D deficiency (25OHD < 10 ng/mL) occurred in 17.6% of patients. Adequate vitamin D levels, defined as serum 25OHD levels > 30 ng/mL, were detected in about one-forth of the CHD patients. Vitamin D status did not differ between males and females nor among CHD groups. Young CHD patients with 25OHD <10 ng/mL did not complain for symptoms of rickets (bowed legs, thickened wrists and ankle, and breastbone projection).

**Figure 1:**
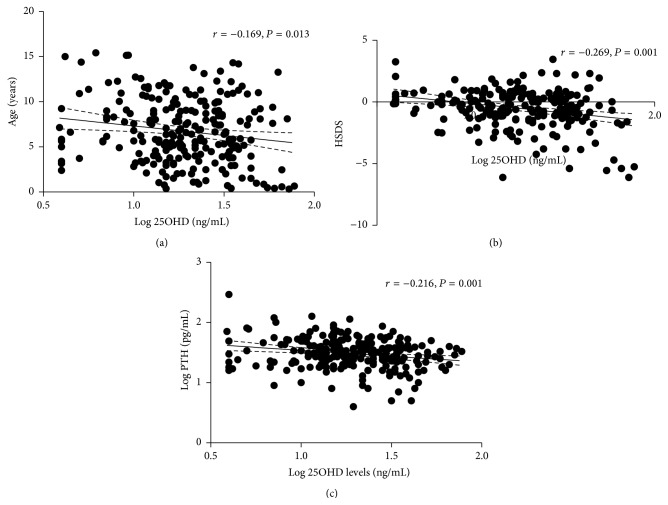
Correlations of serum 25OHD levels with clinical and hormonal markers in young CHD patients. (a) Log 25OHD levels were negatively correlated with age. (b) Log 25OHD levels negatively correlated with height SDS. (c) Log 25OHD levels negatively correlated with Log PTH levels. Best-fit line (continuous lines) and 95% intervals of confidence (dashed lines) were shown.

Serum 25OHD levels negatively correlated with age (*r* = −0.169, *P* = 0.013) (Figure 1(a)) and with height SDS (*r* = −0.269, *P* = 0.001) (Figure 1(b)), also after adjustment for age (*β* = −0.200, *P* = 0.002). Accordingly, CHD patients with severe vitamin D deficiency (<10 ng/mL) were older, heavier, and with higher median PTH levels than patients with an optimal vitamin D status (>30 ng/mL) (Table 1), while serum 25OHD levels did not differ among the three CHD groups identified according the underlying congenital heart defect (Table 2).

### 3.2. Parathyroid Function in Young CHD Patients

Overt hypocalcemia and hypomagnesemia were considered exclusion criteria. Hypercalcemia, defined as elevated levels of serum albumin-corrected calcium and ionized calcium, was not detected in any patients. Hyperparathyroidism, defined as serum PTH levels in the higher quartile (>44 pg/mL), was diagnosed in 56 out of 230 CHD patients (24.0%). Serum PTH levels positively correlated with 25OHD levels (*r* = −0.216, *P* = 0.001; Figure 1(c)) also after adjustment for age (*β* = −0.200, *P* = 0.002). In the present series, renal function was conserved in all CHD patients as confirmed by serum creatinine levels in the normal range.

### 3.3. Effect of Vitamin D Status and PTH Secretion Alterations on Serum NT-proBNP Levels

**Figure 2:**
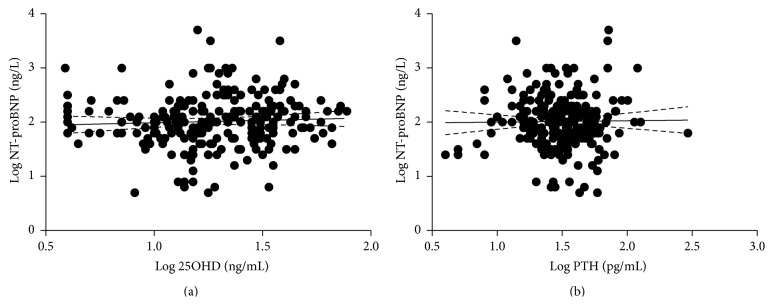
Lack of correlations of serum NT-proBNP levels and 25OHD and PTH levels in young CHD patients. (a) Log 25OHD levels were not correlated with Log NT-proBNP levels. (b) Log PTH levels were not correlated with Log NT-proBNP levels.

Serum NT-proBNP levels ranged from 5.1 to 5529.0 ng/mL in the present series of young CHD patients. Serum NT-proBNP levels did not show any significant correlation with age and did not differ among the different CHD groups (Table 2). Moreover, significant correlations between serum NT-pro-BNP levels and 25OHD (Figure 2(a)) as well as PTH and albumin-corrected calcium failed to be detected (Figure 2(b)).

## 4. Discussion

Circulating NT-proBNP is a cardiac biomarker for diagnosis, prognosis, and therapeutic monitoring. Data available so far in pediatric patients support the NT-proBNP measurement in specific cases [6]. Serum 25OHD and/or PTH levels have been shown to be independently associated with all cause and cardiovascular mortality in adult patients with heart failure [25–28]. Moreover, previous studies reported a negative correlation between serum 25OHD levels and NT-proBNP levels in adult patients with coronary artery diseases and heart failure [27, 29, 30]. Indeed, findings are controversial as an investigation in the setting of adult post-acute myocardial infarction failed in detecting an association between nutritional vitamin D status and NT-proBNP levels [31]. Moreover, a recent interventional study reported any significant effect of oral vitamin D supplementation on circulating NT-proBNP levels in adult peritoneal dialysis patients [32].

In the present series of young CHD patients, we failed in detecting such a relationship between 25OHD and NT-proBNP levels as well as between PTH and NT-proBNP levels, though in the present young CHD cohort vitamin D deficiency and mild hyperparathyroidism frequently occurred.

Data about vitamin D status in young patients with CHD are scanty: Avitabile et al. [33] reported vitamin D deficiency, defined as serum 25OHD levels lower than 20 ng/mL, in 25% of a small series of children and young adults (*n* = 50) with Fontan physiology, while McNally et al. [34] reported hypovitaminosis D in about 40% of 58 young CHD patients.

This is the first study investigating the preoperatively vitamin D status in a consistent series of young patients with various congenital heart defects. In the present series, hypovitaminosis D occurred in half of the patients. Children and adolescents have been reported to be potentially at high risk for vitamin D deficiency [35, 36]. In Europe, where very few foods are fortified with vitamin D, children would appear to be at especially high risk [34]. Indeed, the prevalence of hypovitaminosis D in the present series of CHD young patients was similar to that found in a cohort of Italian healthy children and adolescents [37], suggesting that most of the CHD conditions do not represent a risk factor for the development of hypovitaminosis D in children and adolescents. This point is further supported by the lack of significant differences in median 25OHD levels among the three pathophysiological CHD groups. Moreover, serum 25OHD levels were inversely related to CHD patients' age and height, suggesting, in line with a previous report [38], that older children and adolescents as well as subjects with overweight experience more frequently hypovitaminosis D.

Serum 1,25-dihydroxyvitamin D levels have been suggested to strongly and independently predict cardiovascular mortality in adult patients with chronic heart failure [39]. Therefore, 1,25-dihydroxyvitamin D levels might be more sensitive in detecting the correlation with NT-proBNP levels in young CHD patients; unfortunately, 1,25-dihydroxyvitamin D determinations were not available in the present series of CHD patients.

The serum PTH threshold of 44 pg/mL, corresponding to the upper quartile of the PTH distribution in the young CHD patients, has been chosen to detect mild parathyroid function activation induced by vitamin D deficiency in young subjects with normal renal function [40, 41]. Indeed, PTH levels in healthy children and adolescents have been found to cover a narrower range than the adult values [42]. Considering this PTH threshold, about one-third of hypovitaminosis D young CHD patients showed hyperparathyroidism. Sustained rises in plasma PTH lead to intracellular Ca^2+^ overloading in diverse cells, including cardiomyocytes [43]. In turn, elevated energy ATP stores become depleted and ATPase-dependent Ca^2+^ efflux reduced, together with an induction of oxidative stress that can threaten cardiomyocyte survival. Though the experimental and clinical data suggest an association between NT-proBNP and PTH levels and support their role as predictors of clinical outcomes in adult with cardiovascular diseases, any significant association could be detected between NT-proBNP and PTH levels in young CHD patients.

In conclusion, vitamin D deficiency occurred in half of the young CHD patients and mild hyperparathyroidism could be detected in quarter of the patients. The circulating biomarker NT-proBNP was not related with both vitamin D status and PTH increases, suggesting that calcium metabolism aberrations might not affect circulating NT-proBNP levels in young CHD patients.

## Figures and Tables

**Table 1:** Clinical, biochemical, and hormonal features according to the vitamin D status in young CHD patients.

Markers	Serum 25OHD levels				*P*
	<10.0 ng/mL	10.0–19.9 ng/mL	20.0–30.0 ng/mL	>30.0 ng/mL	
*N*	33	88	48	61	
Age (years)	8.3 (5.6–11.2)	5.8 (3.8–9.0)^*∗*^	7.1 (4.6–9.4)	5.7 (2.8–8.7)^*∗*^	0.02
Male/female	12/21	43/45	23/25	35/26	ns
HSDS	0.02 (−0.54–0.65)	−0.25 (−1.09–0.77)	−0.06 (−1.4–0.31)	−0.83 (−1.6–0.11)^*∗*^	0.002
*W*/*H* (%)	101.5 (91.5–112.5)	97.0 (93.0–107.0)	102.0 (94.0–114.5)	95.0 (88.0–104.5)^§^	0.03
Creatinine (mg/dL)	0.46 (0.37–0.57)	0.41 (0.36–0.51)^*∗*^	0.45 (0.38–0.51)	0.40 (0.32–0.45)^*∗*^	0.01
Alb/calcium (mg/dL)	9.07 (8.80–9.40)	8.97 (8.79–9.25)	9.04 (8.72–9.31)	9.02 (8.80–9.36)	ns
Ionized Ca (mmol/L)	1.19 (1.07–1.25)	1.19 (1.13–1.26)	1.22 (1.08–1.30)	1.21 (1.13–1.30)	ns
Magnesium (mg/dL)	2.08 (1.98–2.14)	2.07 (1.95–2.19)	2.15 (1.98–2.23)	2.05 (1.97–2.15)	ns
Phosphate (mg/dL)	4.86 (4.37–5.38)	5.18 (4.75–5.67)	5.28 (4.77–5.65)	5.11 (4.69–5.67)	ns
PTH (pg/mL)	34.0 (21.5–52.5)	31.0 (24.2–48.7)	31.5 (23.2–43.5)	27.0 (17.5–38.0)^*∗*^	0.003
NT-proBNP (ng/mL)	104.6 (63.2–179.7)	89.1 (43.7–149.2)	129.7 (57.5–283.3)	128.3 (62.9–193.6)	ns

^*∗*^ *P* < 0.05 versus 25OHD < 10 ng/dL; ^§^ *P* < 0.05 versus 25OHD 20–30 ng/dL; ns, not significant; Alb/calcium, albumin-corrected calcium; *W*/*H*, weight/height; HSDS, height standard deviation score; ionized Ca, ionized calcium.

**Table 2:** Clinical, biochemical, and hormonal features according the CHD type in young CHD patients.

Markers	CHD1	CHD2	CHD3	*P*
*n*	153	62	15	
Age (years)	6.4 (4.3–9.2)	7.5 (3.8–10.3)	5.4 (2.6–6.7)	ns
Male/female	65/88	35/27	11/4	ns
HSDS	−0.13 (−1.07–0.59)	−0.54 (−1.42–0.44)	−0.92 (−2.29–0.42)	ns
*W*/*H* (%)	98.0 (92.0–107.0)	98.5 (93.0–110.3)	93.0 (84.0–107.0)	ns
Creatinine (mg/dL)	0.42 (0.36–0.50)	0.44 (0.36–0.55)	0.41 (0.35–0.45)	ns
Alb/calcium (mg/dL)	9.02 (8.80–9.30)	9.06 (8.80–9.33)	8.81 (8.72–9.60)	ns
25OHD (ng/mL)	19.0 (12.8–30.0)	21.3 (12.5–33.4)	17.9 (11.9–30.7)	ns
PTH (pg/mL)	31.0 (22.0–44.5)	28.5 (20.5–41.0)	35.0 (26.0–59.0)	ns
NT-proBNP (ng/mL)	104.6 (60.1–182.5)	109.8 (54.7–200.9)	70.0 (41.0–97.9)	ns

ns, not significant; Alb/calcium, albumin-corrected calcium; *W*/*H*, weight/height; HSDS, height standard deviation score; ionized Ca, ionized calcium.
